# Regulation of Alcohol‐Free and Low‐Alcohol Drinks: Learning From a Comparative Analysis of Eight Countries

**DOI:** 10.1111/dar.70126

**Published:** 2026-02-25

**Authors:** Robyn Burton, Kathryn Angus, Amber Morgan, Rebecca Howell, Nathan Critchlow, Inge Kersbergen, John Holmes, Molly A. Bowdring, Mia Miller, Orratai Waleewong, Aleksi Halme, Carmen Voogt, Niamh Fitzgerald

**Affiliations:** ^1^ Institute for Social Marketing and Health University of Stirling Stirling UK; ^2^ Department of Psychology University of Bath Bath UK; ^3^ School of Health and Related Research University of Sheffield Sheffield UK; ^4^ Stanford Prevention Research Center Stanford University School of Medicine Palo Alto California USA; ^5^ National Drug and Alcohol Research Centre, UNSW Sydney Sydney Australia; ^6^ International Health Policy Program, Ministry of Public Health Nonthaburi Thailand; ^7^ National Supervisory Authority for Welfare and Health Helsinki Finland; ^8^ Trimbos Institute, Netherlands Institute of Mental Health and Addiction Utrecht the Netherlands

**Keywords:** alcohol‐free, low‐strength alcohol, NoLo, non‐alcoholic, regulation, zero alcohol

## Abstract

**Introduction:**

Governance of alcohol‐free and low‐alcohol (No/Lo) drinks has the potential to influence their public health impact. However, regulation remains poorly understood. This study aimed to identify, summarise and compare formal legal frameworks, non‐binding government guidance and recognised self‐regulatory frameworks for the labelling, taxation, licensing and condition of sale and marketing of No/Lo drinks across a diverse set of countries.

**Methods:**

We conducted a desk‐based analysis, supplemented by expert input. Eight case study countries (Australia, Finland, Germany, the Netherlands, Norway, Thailand, the United Kingdom [UK] and the United States of America) were selected for their diversity in geography, alcohol consumption and policy environments. Targeted searches identified documents for determining how No/Lo products were regulated in relation to their labelling, taxation, licensing and conditions of sale and marketing. Data were extracted, tabulated and reviewed for accuracy.

**Results:**

Regulatory thresholds (%ABV) that determine when drinks fall under alcohol legislation vary widely both across and within countries and definitions of No/Lo products are uncommon. For example, drinks can be labelled as alcohol‐free at ≤ 0.05% alcohol by volume (ABV) in the UK, but ≤ 1.15% ABV in parts of Australia. Sales of drinks below defined thresholds generally do not require a premises licence. Marketing restrictions were generally shaped by those for standard alcoholic drinks, although new self‐regulatory guidance has been developed in Australia, the Netherlands and the UK.

**Discussion and Conclusions:**

Governance of No/Lo drinks is fragmented and inconsistent, with definitions and regulatory thresholds varying both across countries and between policy areas within countries.

## 1 Introduction

The term No/Lo drinks generally refers to alcohol‐free, low‐alcohol or lower‐strength alcoholic drinks that share broad similarities (e.g., in taste, appearance or intended use) with standard alcoholic drinks. This sector that has grown considerably over the past decade [1, 2, 3]; however, legal definitions of these products can vary across and within jurisdictions, shaping how they are regulated. This includes who can buy and sell them, where, what taxes apply, how they are labelled and how they can be promoted.

There is little robust, independent evidence on the public health impact of No/Lo drinks. Much of the available research is funded by the alcohol industry, based on small, unrepresentative samples, or uses experiments with limited real‐world generalisability. Market research survey data from the United Kingdom (UK) suggests that some heavy drinkers report drinking alcohol‐free or lower strength drinks to reduce their alcohol intake, but this approach was not as common as other strategies, such as having fewer drinking occasions or drinking smaller serving sizes [4]. In an online hypothetical drink selection task, UK adults were more likely to choose alcohol‐free drinks when they made up a greater share of available options compared to standard alcoholic drinks [5]. In the United States of America (USA), around half of people who drink both alcohol‐free and standard alcoholic drinks believe the former helps them drink less [6].

One larger UK study, supported by a substantial in‐kind contribution from AB InBev, found that introducing 46 new slightly lower‐strength beers (≤ 3.5% alcohol by volume [ABV]) and reformulating 33 existing ones led to reduced purchases of standard alcohol across over 64,000 households [7]. In a related modelling study, members of the same research team estimated that while a 10% reduction in the strength of existing alcoholic drinks averted thousands of deaths annually in six European countries, introducing new beers and wines with an alcoholic strength < 0.5% ABV did not lead to a marked public health impact [7, 8]. In a small randomised controlled trial funded by the brewer Asahi, providing a large volume of free non‐alcoholic beverages reduced alcohol consumption at 12‐week follow‐up by an average of 11.5 g per day, equivalent to roughly one standard drink [9].

No/Lo drinks may reduce harm if they replace stronger alcoholic drinks to a meaningful extent. But the way they are sold, taxed and promoted likely shapes this impact. For example, greater availability of No/Lo drinks in places where alcohol is purchased or consumed may encourage substitution, but promotion or availability of No/Lo drinks in places where alcohol is not normally present may promote the brands that No/Lo drinks often share with alcoholic products. The latter may risk normalising or encouraging consumption of standard alcoholic products, not just No/Lo ones.

To inform debates on how No/Lo drinks might support public health, it is essential to understand how they are governed. This study therefore aimed to identify, summarise and compare formal legal frameworks, non‐binding government guidance and recognised self‐regulatory frameworks on the labelling, taxation, licensing and conditions of sale and marketing of No/Lo drinks across a diverse set of countries. This includes the ABV thresholds at which drinks become subject to alcohol regulations.

## 2 Methods

### 2.1 Design

We undertook a desk‐based review of documents relevant to the governance of No/Lo products, including formal legal frameworks (e.g., legislation and regulation), non‐binding government guidance (e.g., policy documents, advisory notes) and recognised self‐regulatory frameworks (e.g., alcohol industry‐led marketing codes or retailer association agreements) (hereafter ‘governance’ documents). This desk review was supported by supplementary input from subject matter experts in each of the included countries. Rather than applying a single a priori definition of No/Lo drinks, we sought definitions within the governance documents and also examined the ABV thresholds used within each country's regulatory framework to determine when specific labelling, taxation, licensing and conditions of sale and marketing rules apply. In the absence of definitions of No/Lo drinks, these ABV thresholds usually represent the points at which drinks become subject to alcohol legislation within each policy area. Because countries do not use a single consistent alcoholic strength threshold for this purpose, even within their own regulatory systems, we mapped the thresholds embedded in each governance domain rather than defining a single threshold per country.

### 2.2 Country Selection

We selected eight case study countries: Australia, Finland, Germany, the Netherlands, Norway, Thailand, the UK and the USA. These countries were purposively sampled for diversity in terms of geographical spread, per capita alcohol consumption, history of No/Lo consumption/popularity and alcohol policy environment (such as the existence of state‐owned retail monopolies) as fully outlined in Appendix 1. For most countries, the relevant regulations are mainly set at the national level, so we did not consider subnational jurisdictions. However, in the USA, where many of the relevant regulations can be set at both federal and state levels, four states were selected for a more detailed review (Maine, Minnesota, New Jersey and Kentucky) because it was not practical to extract information for every state. This selection was based on the authors' knowledge that these states have diverse regulatory models for alcohol retail (including government‐owned wholesale and/or retail monopolies) and sociodemographic diversity. For Australia, where regulations can also be set at a state level, we extracted information for every state.

### 2.3 Eligible Areas and Types of Governance

Table 1 outlines the eligible governance areas included in our review, selected because they represent the primary levers typically used to regulate standard alcoholic drinks [10, 11]. Eligible documents (source types) included:

- Formal legal frameworks (e.g., legislation and regulations)
- Non‐binding government guidance (e.g., policy documents, advisory notes)
- Recognised self‐regulatory frameworks (e.g., alcohol industry‐led marketing codes or retailer association agreements)

**TABLE 1:** Eligible governance areas documents.

Governance area	In scope	Out of scope
Labelling	Product descriptors (e.g., alcohol‐free, low alcohol); risk and health information labels; product strength information	Nutritional, calorie and allergen labelling
Taxation and pricing	Excise taxes (volumetric, unitary, ad valorem); sales tax; sugar taxes; pricing controls (e.g., fixed price per ml); restrictions/guidelines on price‐based sales promotions	Other taxes (e.g., tax on packaging)
Licensing and conditions sales	Licensing requirements (or guidance) for conditions of sale (e.g., permitted outlets, sales hours); regulation/guidelines on where and when products can be sold; placement and presentation in the retail setting; minimum legal or recommended age of sale/purchase	Regulation on import/export/distribution; legal age requirements for selling or serving No/Lo drinks
Marketing restrictions	Controls on marketing or advertising (e.g., any media visible to the public) regulations/guidance including on claims in advertisements; sponsorship; paid product placement; branded merchandise	—

Documents addressing products that are not typically marketed as alcohol alternatives, such as soft drinks, were excluded. We also excluded governance documents that addressed wider regulatory domains such as nutritional labelling and packaging or environmental taxes.

### 2.4 Identifying Source Documents

We identified documents between June and August 2024 using targeted web searches, snowballing techniques, consultations with subject matter experts and supplementary academic sources. Our goal was to locate documents containing information relevant to the governance areas outlined in Table 1.

We applied a hierarchical approach to document selection. For each governance area and country, we first searched for formal legislation or regulatory frameworks. Where these were unavailable, we sought non‐binding government guidance. In the absence of both, we searched for recognised self‐regulatory documents. Secondary sources were used to supplement our search, particularly where they provided links to primary documents or addressed areas where official documents were lacking.

We used targeted Google searches in English, applying governance‐related keywords (e.g., ‘alcohol‐free marketing’ or ‘alcohol taxation’) alongside country names and legal terminology. We also conducted targeted searches in Google Scholar using similar terms to identify academic articles and reviews relevant to No/Lo alcohol governance that may contain missing information, or signpost to more formal documents. Reference lists of identified documents and academic publications were used to snowball additional sources.

Subject matter experts in each country reviewed the information we identified for accuracy and provided additional documents where gaps existed. Documents were included regardless of publication date, but we used the most recent available where multiple versions existed. When documents were not available in English, we used unofficial translated versions hosted on websites or Google Translate and later verified these translations with native‐speaking experts.

### 2.5 Data Extraction and Review

We extracted information from all relevant documents identified during the source identification stage, using a structured Excel spreadsheet to record data for each of the eligible governance areas outlined in Table 1. To ensure consistency and comparability, decisions regarding the level of information were guided by predefined criteria aligned with the study objectives and refined in consultation with the researchers undertaking the data extraction (K.A., R.B., R.H., A.M.).

Our document review was guided by a set of questions designed to identify how each country classifies and regulates drinks below its standard alcoholic strength. For each policy area, we extracted information on:

- How the jurisdiction defines or describes alcohol‐free, low‐alcohol or lower‐strength drinks (where applicable);
- The specific ABV or other cut‐off points at which particular statutory or non‐statutory requirements apply;
- Any differences in how rules apply across between categories (e.g., beer, wine, spirits);
- Examples that show how the statutory or non‐statutory requirements operate in practice.

Where legal or policy language was particularly specific, we documented excerpts verbatim. More routine or standardised content was summarised for clarity (e.g., ABV thresholds for alcohol‐free definitions). The level of detail extracted for each governance area was determined by its relevance to our analysis and the depth needed for meaningful comparison across countries. For example, policies with significant variation, such as marketing codes, were documented in greater detail to capture nuances.

Following initial extraction, data were summarised into comparative Word tables to support cross‐country analysis. These tables provided a concise overview of each country's position within each governance area, while highlighting key differences and commonalities. Subject matter experts from each included country reviewed the draft tables for accuracy and completeness. They verified extracted information, identified discrepancies and provided additional context or missing documents. Experts also flagged emerging developments and informal practices not yet reflected in formal documentation.

## 3 Results

We report our findings according to the five governance areas outlined in Table 1: labelling; taxation and pricing; licensing and conditions of sale; and marketing restrictions.

### 3.1 Labelling

All countries in our study require products above a certain strength to display the alcohol content (%ABV) on their labels (Table 2). In Finland, Germany, the Netherlands, Norway and the UK, strength labelling is required for products > 1.2% ABV. In Australia, the threshold is ≥ 0.5% ABV and in Thailand and the USA, it's > 0.5% ABV. Across our case study countries, we only identified the UK as having developed guidance on recommended descriptors for alcohol‐free and low strength products, although this has been under review almost continuously since 2018. (Box 1).

**TABLE 2:** A summary of governance documents setting out labelling.

Country	Display of alcohol content	Display of health risk information
	■ Types of regulation	■ Types of regulation
	● Source type	● Source type
Australia	All drinks containing ≥ 0.5% ABV must display alcohol content. For drinks ≤ 1.15% ABV, alcohol content must be written in words to the effect ‘contains not more than X% alcohol by volume’. For drinks ≥ 1.15% ABV, the label must include the alcohol content as a percentage of ABV or mL/100 mL. 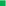 Legislation 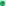 Official [12]	Alcoholic drinks with > 1.15% ABV must include a pregnancy warning label in the form of a pictogram or a pictogram and text: ‘WARNING: Alcohol can cause lifelong harm to your baby’. 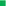 Legislation 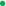 Official [12]
Finland	For drinks containing ≥ 1.2% ABV, the actual alcoholic strength (%ABV) must be listed on the product. 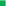 Legislation 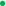 Official [13]	The display of health risk information is not required and we did not find examples of government policy or industry regulation. 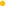 Trusted [14]
Germany	For drinks containing ≥ 1.2% ABV, the actual alcoholic strength must be listed on the product. 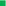 Legislation 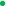 Official [13]	Labels of sweetened alcohol beverages (alcopops) > 1.2% ABV to ≤ 10% ABV must display the following in the same typeface, size and colour as the brand or trade name: ‘Sale is prohibited to persons under 18 years’. 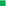 Legislation 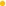 Trusted [15] The display of health risk information for other products is not required however some manufacturers include a pregnancy pictogram on a voluntary basis because of a voluntary corporate scheme. 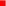 Self‐regulation 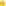 Trusted [16]
Netherlands	For drinks containing ≥ 1.2% ABV, the actual alcoholic strength must be listed on the product. 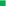 Legislation 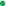 Official [13]	The display of health risk information is not required however some manufacturers include a pregnancy pictogram on a voluntary basis. 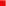 Self‐regulation 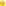 Trusted [17]
Norway	Drinks > 1.2% ABV must display % ABV on the label. 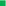 Legislation 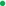 Official [18]	The display of health risk information is under development, exploring statements relating to alcohol's impact on sleep, dementia, heart disease, cancer and liver disease. 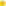 Trusted [19]
Thailand	Drinks ≥ 0.5% ABV must display information about the alcohol content (% ABV). 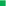 Legislation 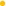 Trusted [20]	Alcoholic drinks ≥ 0.5% ABV must display health risk information, written in Thai, with specific government‐approved wording, such as ‘Liquor drinking may cause cirrhosis and sexual impotency’, ‘Liquor drinking is dangerous to health and causes less consciousness’, ‘Liquor drinking is harmful to you and destroys your family’ and ‘Drunk driving may cause disability or death’. 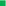 Legislation 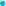 Expert communication
UK	For drinks containing ≥ 1.2% ABV, the actual alcoholic strength by volume (as a percentage) must be listed on the product. 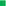 Legislation 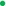 Official [21] Drinks ≤ 1.2% ABV (may be labelled ‘low alcohol’) should display maximum ABV. ‘Alcohol‐free’ should refer to drinks ≤ 0.05% ABV and include ABV or state ‘contains no alcohol’. ‘De‐alcoholised’ should only apply to drinks ≤ 0.5% ABV, with ABV shown. ‘Non‐alcoholic’ should not be used with names typically linked to alcoholic drinks (except for communion wine from unfermented grape juice). 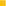 Guidance 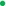 Official [22]	The display of health risk information is not required however the Portman Group (an alcohol‐industry funded body) have developed guidance that recommends manufacturers include a pregnancy logo/message, the low‐risk drinking guidelines and a link to the Drinkaware website (a charity funded by the alcohol industry to provide public health information). This does not explicitly mention No/Lo products. 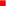 Self‐regulation 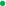 Official [23]
USA	Drinks ≥ 0.5% ABV must display % ABV. If a drink is > 0% and < 0.5% ABV, it is known as ‘non‐alcoholic’ and a statement of alcoholic strength is mandatory if the drink is made with flavours or other non‐beverage ingredients containing alcohol, otherwise optional. 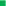 Legislation 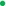 Official [24]	Drinks ≥ 0.5% ABV must display a readily legible mandatory statement: ‘GOVERNMENT WARNING: (1) According to the Surgeon General, women should not drink alcoholic beverages during pregnancy because of the risk of birth defects; (2) Consumption of alcoholic beverages impairs your ability to drive a car or operate machinery, and may cause health problems’. 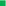 Legislation 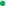 Official [24]

*Note:* Key for types of regulation: 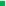 Legislation: legally binding; 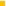 Guidance: non‐statutory, non‐binding government recommendations; 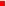 Self‐regulation: industry‐led voluntary codes. Key for source type: 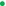 Official: legislation or officially issued policy/guidance; 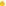 Trusted: unofficial translations of governance documents, peer‐reviewed journals, authoritative reports; 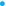 Expert communication: unpublished direct input from national experts; 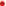 Informal: media articles or other non‐peer‐reviewed sources.

Abbreviation: ABV, alcohol by volume.

BOX 1 **UK consultation on labelling requirements for No/Lo alcohol drinks.**

The UK Government has consulted multiple times on the governance of No/Lo drink descriptors. In 2018, it reviewed how best to label alcohol‐free and low‐strength drinks and chose to retain a voluntary system, defining alcohol‐free as ≤ 0.05% ABV and low strength as ≤ 1.2% ABV [25].

In 2019, the Government committed to reviewing the possibility of raising the alcohol‐free descriptor to ≤ 0.5% ABV to align with other European countries [26]. An evidence review published by the Office for Health Improvement and Disparities in 2023 assessed the potential impacts of this change for pregnant women, drivers and individuals in recovery from alcohol dependence [27]. The review noted the limited size of the alcohol‐free market in the UK and highlighted that the UK's definition is inconsistent with international norms, considered to be ≤ 0.5% ABV.

Following this in September 2023, a second consultation was launched to gather feedback on whether descriptors should change, and whether No/Lo drinks should include low risk drinking guidelines, health warnings or age restrictions [28]. A government response is usually expected within 12 weeks [29], but as of writing, no update has been issued. In July 2025, the Department for Health and Social Care published the 10‐year Health Plan for England which included the commitment to ‘consult on changing the upper strength threshold at which a drink may be described as alcohol free to 0.5% ABV’. Alongside this, they stated that they will ‘explore options to restrict access to No/Lo products so they are treated in the same way as all alcohol products, including banning sales to under 18 year‐olds [30]’.

Health risk information on labels is less consistent. Mandatory health warnings are required in Australia (≥ 1.15% ABV), Thailand (≥ 0.5% ABV) and the USA (≥ 0.5% ABV), largely relating to information about drinking during pregnancy, driving and when underage (Table 2). In contrast, Finland, Germany, the Netherlands, Norway and the UK have no legal requirement to display warnings, though voluntary measures such as pregnancy pictograms are sometimes used.

### 3.2 Taxation and Pricing

Excise tax thresholds based on % ABV vary across countries and sometimes by drink type (Appendix 2: Table A1). Most countries begin taxing from > 0.5% ABV, including the USA, Thailand, and for beer in Finland, Germany and the Netherlands. Higher thresholds are used in Norway (> 0.7% ABV), Australia (> 1.15% ABV) and the UK (> 1.2% ABV). In Finland, Germany and the Netherlands, beers are taxed from > 0.5% ABV, but other drink types from > 1.2% ABV. For drinks below these thresholds, most countries apply ad valorem general sales taxes, ranging from 7% of retail price in Thailand to 25.5% in Finland. We did not find examples of any other pricing controls for products below the excise tax threshold.

### 3.3 Licensing and Conditions of Sales

A licence is typically required to sell drinks classified as alcoholic, but the definition of what counts as alcoholic for this purpose varies across countries. The most common threshold is > 0.5% ABV, used in the Netherlands, Thailand and the UK, and some states in Australia and the USA (Table 3). Finland and Norway apply higher thresholds of > 2.8% ABV and > 2.5% ABV respectively. Germany is an outlier as no licence is required for the retail sale of alcohol, although other laws still apply in relation to health, safety and youth protection. In all countries, products below these ABV thresholds may be sold without a licence.

**TABLE 3:** A summary of regulation for licensing and conditions of sale.

Country	The ABV threshold at which a licence is required to sell a product	Minimum purchase age for products above the licensing threshold	Types of regulation	Source type
Australia	> 0.5% to > 1.15% ABV (depending on state)	18 years	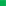 Legislation and 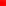 Self‐regulation (voluntary refusal of NoLo to minors)	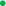 Official [31, 32, 33, 34, 35, 36, 37, 38] and 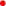 Informal [39]
Finland	> 2.8% ABV	18 years^b^	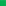 Legislation and 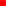 Self‐regulation (voluntary refusal of NoLo to minors)	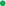 Official [40] and 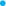 Expert communication
Germany	No licence required. Drinks < 0.5% ABV are not age restricted. Drinks between 0.5% and < 1.2% ABV can be purchased from age 16, ≥ 1.2% ABV from age 18^a^	16 years	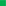 Legislation	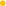 Trusted [41] and 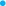 Expert communication
Netherlands	> 0.5% ABV	18 years	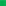 Legislation	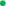 Official [42]
Norway	> 2.5% ABV	18 years	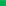 Legislation	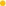 Trusted [43]
Thailand	> 0.5% ABV	20 years	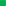 Legislation	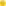 Trusted [20]
UK	> 0.5% ABV	18 years	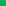 Legislation and 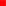 Self‐regulation (voluntary refusal of NoLo to minors)	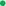 Official [44, 45, 46, 47]
USA	Varies by state (see Appendix 3: Table A2)			

*Note:* Key for types of regulation: 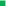 Legislation: legally binding; 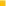 Guidance: non‐statutory non‐binding government recommendations; 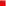 Self‐regulation: industry‐led voluntary codes. Key for source type: 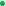 Official: legislation or officially issued policy/guidance; 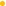 Trusted: unofficial translations of governance documents, peer‐reviewed journals, authoritative reports; 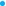 Expert communication: unpublished direct input from national experts; 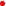 Informal: media articles or other non‐peer‐reviewed sources.

Abbreviation: ABV, alcohol by volume.

^a^ In Germany, there is no licence required for the production, wholesale or retail sales of alcoholic beverages specifically, but there are laws non‐specific to alcohol which affect and essentially regulate these activities [48].

^b^ The licensing threshold is > 2.8% ABV, but the minimum purchase age applies to beverages > 1.2% ABV. Restaurants and public houses are required to obtain a licence which comes with obligations and regulations, namely around food safety and youth protection laws.

Similarly, minimum legal purchase ages apply only once a drink crosses the jurisdiction's alcohol threshold. This means that drinks below the licensing threshold are exempt from age restrictions. For example, in the UK and the Netherlands, under‐18 s can buy drinks ≤ 0.5% ABV, increasing to ≤ 0.7% ABV in Norway, ≤ 1.15% ABV in Australia and ≤ 1.2% ABV in Finland. In Germany, drinks < 0.5% ABV are not age‐restricted and minors under 16 can buy drinks between 0.5% and < 1.2% ABV. In the USA, no federal restrictions exist for minimum legal purchase age and this varies by state, 23% of which have state‐wide age restrictions for alcohol‐free drinks (Appendix 3: Table A2). Despite these restrictions, we identified voluntary practices in Australia, Finland, the UK and the USA, where some retailers choose not to sell No/Lo drinks to minors, even when legally permitted.

### 3.4 Marketing Restrictions

Marketing regulations for No/Lo drinks vary widely across countries and are often shaped by how standard alcoholic drinks are governed. While advertising for standard alcoholic beverages is broadly prohibited in Norway and Thailand, and heavily restricted in Finland, rules for No/Lo drinks still differ significantly between these countries.

In Norway, all alcohol advertising is banned, including for No/Lo drinks, unless the product contains exactly 0% ABV and does not share branding with an alcoholic beverage. Thailand prohibits advertising for drinks > 0.5% ABV and plans to extend the ban to any drinks, including soft drinks such as water, that share branding with alcoholic products. In Finland, drinks ≤ 1.2% ABV may be advertised like any other food, provided the advertising is not considered indirect promotion of alcohol. Advertising is restricted for products that share branding with an alcoholic drink ≤ 22% ABV and prohibited if the associated drink is > 22% ABV.

By contrast, Australia, the Netherlands and the UK permit advertising of both standard and No/Lo alcohol products across all media, though with varying restrictions (see Box 2). In the USA, there are no federal marketing restrictions for alcohol‐free drinks and no state‐level rules were identified in the four states examined (Appendix 4). Germany also does not regulate the marketing of alcohol‐free drinks, although standard alcohol marketing restrictions apply to drinks > 0.5% ABV.

BOX 2 **Examples of marketing restrictions for No/Lo drinks in Australia, the Netherlands and the UK.**

**The UK**

In 2022, the Committee of Advertising Practice, a self‐regulatory body, consulted on extending marketing codes to alcohol alternatives on broadcast and non‐broadcast media [49]. Previously, alcohol‐free drinks (≤ 0.5% ABV) were largely exempt. The new code, announced late 2023, applies from May 2024 [50].

Under the revised code, advertising for alcohol‐free drinks (beverages with an ABV at or below 0.5% and referred to as ‘alcohol alternatives’) should not target or have particular appeal to individuals < 18 years, appear in media where > 25% of the audience is < 18 years or ‘imply, condone, or encourage immoderate, irresponsible, or anti‐social drinking’ [51, 52]. All advertisements should include the drink's %ABV.

Under the code, alcohol alternatives may be advertised as preferable due to their lower alcohol content, unlike standard alcoholic drinks, which can only make factual strength statements (without suggesting it is preferable due to its strength or intoxicating effect). If an advert indirectly promotes a standard alcoholic product, usual alcohol advertising rules apply. However, alcohol alternatives sharing the same brand as an alcoholic drink are exempt if the primary focus is clearly on the alcohol alternative. Advertisements for alcohol alternatives can also depict consumption in contexts where drinking alcohol would be inappropriate (e.g., before driving, or in the morning), provided it is clear the product is alcohol‐free [52].

**Netherlands**

The Dutch Advertising Code for Alcohol‐Free and Low‐Alcohol Beer (a voluntary industry code) came into effect in April 2024 [53]. This code stipulates that advertising of non‐alcoholic variants of an alcoholic beverage (with an alcohol content of up to 0.5% ABV) may not be aimed at people < 18, pregnant women or drivers [53, 54]. Non‐alcoholic variants are not defined according to % ABV, and instead as ‘a beverage intended for human consumption, not being an alcoholic beverage, where reference is made to an alcoholic beverage through the name, brand, product design, and/or marketing of that alcoholic beverage’. Additionally, advertisements for non‐alcoholic versions of alcoholic drinks cannot be directed at an audience where > 25% are minors. Although the code has not been evaluated, a realist review speculates it is unlikely to be effective, based on prior assessments of similar voluntary codes [55].

**Australia**

Australia's Responsible Alcohol Marketing Code, an industry‐led, voluntary standard, applies to both standard alcoholic drinks and alcohol alternatives (a beverage that is at or less than 0.5% ABV) [56]. Key elements include ensuring marketing does not encourage excessive consumption or target or appeal to minors, and that ads are placed in media with at least an 80% adult audience. Furthermore, communications for alcohol alternatives should clearly identify the product as such and adhere to similar standards if they associate the product with alcohol consumption, such as through branding, imagery or messaging that mimics alcoholic beverages.

## 4 Discussion

This study aimed to identify, summarise and compare formal legal frameworks, non‐binding government guidance and recognised self‐regulatory frameworks on the labelling, taxation, licensing and conditions of sale, and marketing of No/Lo drinks across a diverse set of countries. A central finding is that, with limited exceptions, countries do not regulate a distinct category of No/Lo drinks. Instead, rather than defining ‘alcohol‐free’ or ‘low‐alcohol’ products as separate legal categories, most jurisdictions rely on ABV thresholds to define standard alcoholic drinks within each regulatory domain. These thresholds then determine the point at which specific rules or guidance begin to apply or no longer apply. These thresholds vary not only between jurisdictions, but also across policy areas within the same country. Except for Thailand, none of our case study countries apply a uniform threshold across all policy areas considered in this paper. While previous research has noted international inconsistencies in ABV thresholds used to classify No/Lo drinks [57], the lack of coherence within countries means that the regulations are difficult to straightly summarise. Researchers and policymakers should therefore avoid assuming or suggesting that No/Lo drinks have a single, consistent definition within any country.

In the countries included in our study, lower strength ABV products that would generally be considered alcohol‐free, and to a lesser extent those considered to be low‐alcohol drinks, are often exempt from regulations or guidelines that apply to standard alcoholic beverages, including those related to labelling, taxation, licensing and conditions of sale and marketing. This is not necessarily due to deliberate exclusion, but rather a byproduct of longstanding legislation that uses ABV thresholds to define what counts as ‘alcohol’ and, therefore by omission, what does not. These definitions, often established before alcohol‐free products became more widely available, determine what is and what is not regulated, and, to some extent, what any regulations look like. For example, licensing laws across the countries in our study often define alcohol using an ABV threshold, meaning alcohol‐free drinks falling below this threshold can be sold in unlicensed premises and are not subject to the same availability restrictions, like sales hours or minimum age of sale. These products are also usually tax‐exempt, with Thailand as the exception of all the countries in our study. Similarly, although five of the eight countries require or recommend health information on alcoholic products, these warnings do not extend to drinks that fall below a certain ABV threshold. Marketing was the only governance area where we found specific guidance for alcohol‐free products having been developed to fill this regulatory gap, though notably, only Norway and Thailand have statutory restrictions on marketing of shared‐branded No/Lo products. In Australia, the Netherlands and the UK, only non‐statutory, self‐regulatory codes exist regarding the marketing of No/Lo drinks (Box 2). The reliance on ABV thresholds to determine when specific rules apply may work well for areas such as taxation, but in others, such as marketing, the ABV alone may not capture important aspects of how products are presented or perceived that have consequences for public health, given the shared branding of standard alcohol and alcohol‐free versions. While our study does not aim to assess the effectiveness of current regulatory approaches or prescribe alternatives, these observations highlight that the suitability of ABV as an organising principle varies by domain, and future work could explore whether No/Lo drinks should be treated in the same way, or differently to, standard alcoholic drinks would better support harm‐minimisation goals.

One important oversight across the countries included in our study is the minimum legal purchase age. Because licensing laws define alcohol based on strength, No/Lo drinks below the legal threshold can be purchased by minors. While governments often state that No/Lo drinks are intended for adults and some retailers impose voluntarily age restrictions (e.g., in Australia, Finland and the UK), formal legal frameworks are lacking [28, 44]. Governments have not generally clarified the rationale for treating these products as adult‐only, but concerns include exposure of minors to the taste of alcohol and alcohol‐related practices and the potential for No/Lo drinks to act as a gateway to alcohol use or reinforce social norms around drinking [58, 59, 60]. The limited research to date provides unclear findings [61, 62, 63], though some evidence suggests that young people see No/Lo drinks primarily as tools to avoid rather than move towards alcohol consumption [64].

No/Lo drinks are allowed to be sold in a wider range of retail settings than standard alcoholic drinks across the countries included in our study. This could facilitate substitution, helping individuals to reduce their consumption by offering a widely accessible alternative to standard alcoholic drinks. Alternatively, it could normalise drinking behaviours in contexts or scenarios where alcohol consumption is not typical, potentially expanding the contexts in which drinking alcohol is viewed as socially acceptable.

While No/Lo drinks may support alcohol reduction at the individual‐level, their unrestricted marketing may raise concerns. These products often share branding, packaging and visual design with standard alcoholic drinks [65, 66, 67]. This creates a risk of surrogate marketing or brand stretching, whereby alcohol brands are promoted in media or spaces where direct advertising of alcoholic beverages would otherwise be restricted [66]. Whether consumers, particularly young consumers, can distinguish between No/Lo and standard alcoholic brands is unclear. Research in Thailand among university students (aged 20–24) found that brand extensions by alcohol companies increased brand awareness, recognition and familiarity of alcohol brands [68]. While substitution may occur if consumers perceive no/lo drinks as appropriate replacements, qualitative work in the UK suggests that alcohol‐free drinks are often marketed and perceived to be relevant for drivers, pregnant women, dieters, sports people and people who were under age, rather than as universal substitutes for alcohol [69]. Other research suggests that some advertisements for no/lo drinks promote use in new settings, e.g., at work or while driving, rather than as replacements in existing drinking occasions [70, 71]. This underscores the tension between no/lo drinks as a tool for harm reduction and as products that may reinforce or expand drinking norms. Increasing familiarity with these products through shared branding may support substitution but could also carry risks if the branding extends the visibility of alcohol into restricted settings.

Compared to the size of the alcohol market, the size of the no/lo market is small, and its current and long‐term impact on public health remains unknown, however it is growing rapidly. Given the accompanying rapid growth in availability of no/lo drinks and related marketing, to which young people are also exposed, public health policy stakeholders should devise a more coherent policy framework covering all of these policy domains. Governance of no/lo drinks must strike a balance between encouraging substitution and managing unintended consequences. If these products are shown to reduce consumption of standard alcoholic drinks, pairing restrictions on alcohol sales, with increased availability and visibility of no/lo drinks could offer meaningful public health benefits. However, without stronger regulatory oversight, the expansion of no/lo marketing and availability may also reinforce alcohol brands, normalise drinking behaviours and introduce new risks. Until more research is available, a precautionary approach is likely warranted. However, the growing market presence of no/lo drinks warrants continued attention. Further studies could support this by monitoring consumption patterns, and drivers of consumption of both no/lo and alcohol products under different regulatory regimes. This could include examining the impacts of exposure to marketing and availability of no/lo drinks and relevant parent alcohol brands, as well as different tax thresholds. Researchers could also seek to explore public consensus on no/lo regulation in the public interest.

The emerging focus on no/lo drinks should not detract from the need to implement the World Health Organization’s so‐called “best buy” policies which have proven effectiveness for preventing and reducing alcohol‐related harm. The lack of statutory regulations for no/lo drinks may reflect gaps in regulation of standard alcoholic drinks more broadly. For example, while no countries required health information or warnings on alcohol‐free drinks, five of the eight also do not require health information on standard alcoholic drinks. This raises the question of whether the governance gaps observed for no/lo drinks are not exceptional, but rather symptomatic of wider weaknesses in alcohol regulation regarding labelling and marketing. In countries where alcohol marketing is ubiquitous, marketing of no/lo drinks is also typically governed through self‐regulatory industry codes, which lack independent enforcement and may not be effective at protecting public health [72].

### 4.1 Strengths and Limitations

Our study is the first to explore approaches to regulating no/lo products, drawing on international information across eight countries in four continents. Our search methods were supplemented with expert verification, increasing the identification and accuracy of description of the included approaches. Nonetheless, our study has several limitations. While all attempts were made to identify relevant governance, it is possible that some were overlooked and there are other restrictions in place which are not reported here. This may be particularly true for voluntary agreements which do not feature in official legislation or policy documents. Although we identify and document policy, we do not make any assessment of policy implementation, namely what the policy says and what is happening in practice (e.g., degree of compliance). Stricter regulation may be more acceptable in countries with well‐established state regulation of alcohol, such as those with state monopolies on alcohol retail. We did not include or seek to identify possible loopholes that exist in restrictions. We cannot generalise our findings to other countries, which may take different approaches to regulating no/lo drinks.

## 5 Conclusion

The regulation of no/lo drinks is highly variable between and within the eight diverse countries examined in this study, with many current regulations based on legacy definitions of what constitutes an alcoholic drink. Alcohol‐free drinks, and to a lesser extent, low‐alcohol drinks, are often exempt from rules governing standard alcohol, including in key areas like labelling, taxation, marketing, and licensing and conditions of sale. In the eight countries considered, most regulatory frameworks have not been updated in the light of the rise in popularity, availability and marketing of no/lo beverages, and are not designed to prevent consequent harms or facilitate benefits. Future research should explore the effects and unintended consequences of different regulatory approaches.

## Author Contributions

Conceptualisation: R.B., K.A., A.M., R.H., J.H., N.F. Methodology: R.B., K.A., A.M., R.H. Formal analysis: R.B., K.A., A.M., R.H., M.A.B., M.M., O.W., A.H., C.V. Investigation: R.B., K.A., A.M., R.H. Writing original draft: R.B., K.A., A.M., R.H. Writing reviewing and editing: R.B., K.A., A.M., R.H., N.C., I.K., J.H., M.A.B., M.M., O.W., A.H., C.V., N.F. Visualisation: R.B., K.A., A.M., R.H., M.A.B., M.M., O.W., A.H., C.V. Funding acquisition: J.H., N.F., N.C., I.K.

## Funding

This study was funded by the National Institute for Health and Care Research (NIHR) Public Health Research programme (NIHR135310). The views expressed are those of the author(s) and not necessarily those of the NIHR or the Department of Health and Social Care. This work was made possible in part by a National Institute on Alcohol Abuse and Alcoholism (NIAAA)‐funded K99 award (1K99AA031716) to M.A.B.

## Conflicts of Interest

M.A.B. has consulted for a technology company aimed at helping people to reduce their alcohol use. N.C. was on the board of directors at Alcohol Focus Scotland between 2017 and 2022. J.H., I.K. and N.F. have received research funding from Alcohol Change UK (ACUK) for an unrelated alcohol research project (N.F. and J.H.) and for a project on young people's use of alcohol‐free and low‐alcohol drinks (J.H., I.K.). ACUK has several commercial partnerships for its Dry January campaign, including Walkers Crisps (A PepsiCo brand) and Lucky Saint, an independent brewer of alcohol‐free beers which acquired a pub that sells alcohol in 2023 and became an associate member of the alcohol industry responsibility body the Portman Group in 2025. The partnership with Lucky Saint provides ACUK with < 0.6% of its annual income. The remaining authors declare no conflicts of interest.

## Data Availability

The data that support the findings of this study are available from the corresponding author upon reasonable request.

## Appendix 1 Alcohol Per Capita Consumption Age 15+ Years 2019 (Three‐Year Average of 2017, 2018, 2019) [73]

### Sampling Notes

The project team is based in the UK, and the funding for this project comes from the UK National Institute for Health and Care Research, and so sampling was focused primarily on high‐income countries.

Australia and the USA were included as large jurisdictions outside of Europe with diverse, multi‐level alcohol regulatory systems, which may yield interesting insights into No/Lo policy approaches. In Australia, alcohol governance is shared across federal and state/territory levels, with federal law covering areas such as taxation and labelling, and states/territories responsible for licensing and conditions of sale. A similar division of regulatory responsibility is seen in the USA. Both have some similarities in alcohol policy and culture to the UK, and also some differences.

The Netherlands and Germany were selected as they were known to be European countries in which No/Lo drinks have been popular over a much longer period than in the UK, and therefore No/Lo policy approaches may be longer established. The Netherlands was included also partly due to evidence of higher levels of No/Lo consumption among young people.

Finland and Norway were included as examples of Nordic countries with state‐owned alcohol retail monopolies for beverages above a certain ABV, whilst permitting lower strength products to be sold in grocery stores. Norway was of particular interest as it was known to the team that it had policies restricting shared‐brand No/Lo advertising.

Thailand (an upper‐middle income country) was included to ensure some geographical diversity and because it was known to have some policy and research interest in this topic.

## Appendix 2

**TABLE A1:** Summary of regulation related to the taxation of No/Lo drinks.

Country	Excise tax threshold (% ABV)	Sales tax: % ABV threshold (ad valorem rate)	Types of regulation	Source type
Australia	> 1.15% ABV	> 1.15% ABV (10%)	Legislation	Official [74]
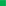
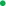
Finland	> 0.5% ABV (beer) > 1.2% ABV (other drinks)	≥ 0% ABV (25.5%)	Legislation	Official [75]
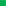
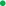
Germany	> 0.5% ABV (beer) > 1.2% ABV (other drinks)	≥ 0% ABV (19%)	Legislation	Official [75]
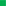
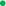
Netherlands	> 0.5% ABV (beer) > 1.2% ABV (other drinks)	≥ 0% ABV (21%)	Legislation	Official [75]
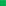
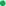
Norway	> 0.7% ABV	≥ 0% ABV (15%)	Legislation	Official [76]
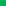
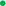
Thailand	> 0.5% ABV	≥ 0% ABV (7%)	Legislation	Expert communication
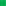
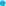
UK	> 1.2% ABV	≥ 0% ABV (20%)	Legislation	Official [77]
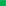
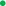
USA	> 0.5% ABV	≥ 0% ABV (10%)	Legislation	Official [78]
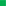
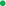

*Note:* Key for types of regulation:  Legislation: legally binding;  Guidance: non‐statutory, non‐binding government recommendations;  Self‐regulation: industry‐led voluntary codes. Key for source type:  Official: legislation or officially issued policy/guidance;  Trusted: unofficial translations of governance documents, peer‐reviewed journals, authoritative reports;  Expert communication: unpublished direct input from national experts;  Informal: media articles or other non‐peer‐reviewed sources.

Abbreviation: ABV, alcohol by volume.

## Appendix 3

**TABLE A2:** Alcohol regulators' state‐wide definitions of alcohol and policies about non‐alcoholic beverage sales to minors in the USA. Local ordinances may have stricter definitions of alcohol and non‐alcoholic beverages. Adapted from [79].

State	Definition of alcoholic/non‐alcoholic used in licensing legislation	Sales of non‐alcoholic drinks permitted to minors (Y/N)	Details	Source
Alabama	Alc: ≥ 0.5%	Y	Not regulated	Alabama Alcoholic Beverage Control Board
Alaska	NAB: < 0.5%	Y	Not regulated	Alaska Alcohol and Marijuana Control Office
Arizona^a^	Alc: > 0.5%	Y	Not regulated	Arizona Alcohol Traffic and Firearms
Arkansas^a^	Alc: > 0.5%	Y	Not regulated	Arkansas Alcohol Beverage Control
California^a^	Alc: ≥ 0.5%	Y	Not regulated	California Department of Alcohol Beverage Control
Colorado^a^	Alc: > 0.5%	Y	Not regulated	Colorado Department of Revenue–Liquor Enforcement Division
Connecticut^a^	Alc: ≥ 0.5%	Y	Not regulated	Connecticut Department of Consumer Protection
Delaware	Alc: ≥ 0.5%	Y	Not regulated	Delaware Office of the Alcoholic Beverage Control Commissioner
District of Columbia^a^	Alc: ≥ 0.5%	Y	Not regulated	District of Columbia Alcoholic Beverage Regulation Administration
Florida	Alc: > 0.00%	N if > 0.00%	If a beverage has an ABV > 0.00%, then it is regulated like alcohol (i.e., sales not permitted to minors < 21 years). Sale to minors is permitted for NABs that are exactly 0.00%	Florida Division of Alcoholic Beverages and Tobacco
Georgia^a^	Alc: Defined by how it is produced (not ABV)	N	Any NAB that is produced in the same way as its alcoholic counterpart is regulated as alcohol (i.e., sales not permitted to minors < 21 years)	Georgia Department of Revenue Alcohol and Tobacco Tax Division
Hawaii^a^: Alcohol is regulated by county (below)				
Honolulu^a^	NAB: < 0.5%	Y	Not regulated	Liquor Commission City and County of Honolulu
Hawaii^a^	NAB: < 0.5%	N	Retailers are not permitted to sell NABs to minors < 21 years	Department of Liquor Control County of Hawaii
Kauai^a^	Alc: ≥ 0.5%	Y	Not regulated	Department of Liquor Control County of Kauai
Maui^a^	Response not received	—	—	—
Idaho^a^	Alc: Defined by how it is produced (not ABV)	N	Any NAB that is produced in the same way as its alcoholic counterpart is regulated as alcohol (i.e., sales not permitted to minors < 21 years)	Idaho State Liquor Division and Alcohol Beverage Control
Illinois^a^	Alc: > 0.5%	Y	Not regulated	Illinois Liquor Control Commission
Indiana^a^	Alc: ≥ 0.5%	Y	Not regulated	Indiana Alcohol and Tobacco Commission
Iowa	Alc: > 0.5%	Y	Not regulated	Iowa Alcoholic Beverages Division
Kansas	Alc: > 0.00%	N if > 0.00%	If a beverage has an ABV > 0.00%, then it is regulated like alcohol (i.e., sales not permitted to minors < 21 years). Sale to minors is permitted for NABs that are exactly 0.00%	Kansas Department of Revenue Alcohol Beverage Control
Kentucky^a^	Alc: Anything sold as, or under any name commonly used for, alcoholic beverages, whether containing any alcohol or not	N	Sales of NA beer, wine or spirits are not permitted to minors < 21 years	Kentucky Alcoholic Beverage Control Department
Louisiana	Alc: ≥ 0.5%	Y	Not regulated	Louisiana Department of Revenue Alcohol and Tobacco Control Office
Maine^a^	NAB: < 0.5%	N	Administrative violation for retailers to sell NABs to minors < 21 years	Maine Bureau of Alcoholic Beverages and Lottery Operations
Maryland^a^	Alc: ≥ 0.5%	Y	Not regulated	Maryland Alcohol, Tobacco and Cannabis Commission
Massachusetts^a^	Alc: ≥ 0.5%	Y	Not regulated	Massachusetts Alcoholic Beverages Control Commission
Michigan^a^	NA beer: Defined by how I is produced; NA wine/liquor: < 0.5%	N if < 18 years for NA beer; Y for NA wines/liquors	Beer (regardless of alcohol content), if it is a cereal beverage, may not be sold to people < 18 years. No policy for NA wine/liquors	Michigan Liquor Control Commission
Minnesota	Alc: > 0.5%	Y	Not regulated	Minnesota Alcohol and Gambling Enforcement
Mississippi^a^	Malt beverage: ≤ 10.3% ABV; light spirits: ≤ 7.5% ABV; light wine: ≤ 6.25% ABV	N	Retailers not permitted to sell beverages as defined to minors < 21 years	Department of Revenue–Beer Department
Missouri^a^	Alc: > 0.5%	Y	Not regulated	Division of Alcohol and Tobacco Control
Montana^a^	Alc: > 0.5%	Y	Not regulated	Montana Alcohol Beverage Control Division
Nebraska	Alc: ≥ 0.5%	N for NA beer; Y for NA wines/liquors	NA beer is regulated the same as beer (i.e., sales not permitted to minors < 21 years); no policy for NA wines/liquors	Nebraska Liquor Control Commission
Nevada^a^	Alc: ≥ 0.5%	Y	Not regulated	Nevada Legislative Counsel Bureau
New Hampshire^a^	Alc: ≥ 0.5%	Y	Not regulated	New Hampshire State Liquor Commission
New Jersey^a^	Alc: > 0.5%	Y	Not regulated	Division of Alcoholic Beverage Control
New Mexico^a^	NAB: < 0.5%	Y	Not regulated	New Mexico Regulation and Licensing Department
New York^a^	NAB: < 0.5%	Y	Not regulated	New York State Liquor Authority Division of Alcoholic Beverage Control
North Carolina^a^	Alc: ≥ 0.5%	Y	Not regulated	North Carolina Alcoholic Beverage Control Commission
North Dakota	Alc: ≥ 0.5%	Y	Not regulated	North Dakota Office of the State Tax Commissioner
Ohio^a^	NAB: < 0.5%	N if < 18 years	NABs may not be sold people < 18 years of age	Ohio Liquor Control
Oklahoma^a^	Alc: > 0.5%	Y	Not regulated	Oklahoma Alcoholic Beverage Law Enforcement Commission
Oregon^a^	Alc: > 0.5%	Y	Not regulated	Oregon Liquor Control Commission
Pennsylvania	NAB: > 0.00%, ≤ 0.5%	N if > 0.00%	If a beverage has an ABV > 0.00%, then sales are not permitted to minors < 21 years. Sale to minors is permitted for NABs that are 0.00%	Pennsylvania Liquor Control Board
Rhode Island	Alc: ≥ 0.5%	Y	Not regulated	Rhode Island Liquor Enforcement and Compliance
South Carolina^a^	NA and non‐intoxicating beverages: Beers, ales, porters, and similar malt or fermented beverages < 5% ABV and wines < 21% ABV	N	Sale of NABs are not permitted to minors < 21 years	South Carolina Department of Revenue and Taxation
South Dakota^a^	Alc: > 0.5%	Y	Not regulated	South Dakota Department of Revenue
Tennessee^a^	Alc: ≥ 10.1%	Y	Not regulated	Tennessee Alcoholic Beverage Commission
Texas^a^	Alc: ≥ 0.5%	Y	Not regulated	Texas Alcoholic Beverage Commission
Utah	Alc: ≥ 0.5%	Y	Not regulated	Utah Department of Alcoholic Beverage Control
Vermont^a^	Alc: ≥ 1.0%	Y	Not regulated	Vermont Department of Liquor and Lottery
Virginia^a^	NAB: < 0.5%	Y	Not regulated	Virginia Alcoholic Beverage Control Authority
Washington	Alc: ≥ 0.5%	Y	Not regulated	Washington State Liquor and Cannabis Board
West Virginia^a^	Alc: > 0.5%	Y	Not regulated	West Virginia Alcohol Beverage Control Administration
Wisconsin^a^	Alc: ≥ 0.5%	Y	Not regulated	Wisconsin Department of Revenue
Wyoming^a^	Alc: ≥ 0.5%	Y	Not regulated	Wyoming Liquor Control

*Note:* Author (M.A.B.) contacted each state's alcohol regulatory board between 12/2023 and 1/2024, and each source listed provided a response confirming their state policy. Nevada's response was from the Legislative Counsel Bureau as there is not a state agency that regulates alcohol sales. Alcohol is regulated by county in Hawai‘i, thus, county regulators were contacted.

Abbreviations: ABV, alcohol by volume; Alc, alcohol; NAB, non‐alcoholic beverage (‘non‐alcoholic’ is the term used in the USA as opposed to ‘alcohol‐free’).

^a^ Personal communication with the state's department of health confirmed that their department does not regulate sale of NABs (responses were not received from states without this superscript).

## Appendix 4 A Detailed Overview of Regulations, Guidelines and Policies on No/Lo Alcohol Drinks Across Selected US States

Since not all alcohol legislation is set at a state‐level, we further explored No/Lo regulation, guidance, and policy in four US states: Maine, Minnesota, New Jersey and Kentucky. These states were chosen in discussion with a topic expert in the USA and represent a range of regulatory models (including monopolies) and sociodemographic diversity.

While states have primary authority over alcohol regulation, both the federal and local government play an important role in ensuring an efficient alcohol regulatory system [80]. Alcohol regulation involves coordination and communication between federal, state and local government agencies. At the federal level, the Alcohol and Tobacco Tax and Trade Bureau has primary responsibility for enforcing federal alcohol laws, overseeing production, importation, wholesale distribution, labelling and advertising. The Federal Trade Commission addresses concerns about the effects of alcohol marketing on youth. The Food and Drug Administration is responsible for enforcing the federal food and drug law. The Federal Communications Commission is responsible for communications. At the state level, most states have a state agency that has primary regulatory authority over alcohol, which oversees the sale, distribution and administrative enforcement of the state alcohol code.

### Maine

Maine has a control model, wherein the government directly controls the distribution and sale of alcohol. In Maine [80]. Any product that contains < 0.5% ABV is legally considered to be a non‐alcoholic drink and is therefore exempt from the regulations set out in the licensing legislation (expert communication). Therefore, a vendor does not need a licence to sell non‐alcoholic drinks, and they can be sold 24 h a day, 7 days a week. The only restriction on non‐alcoholic drinks is age. If a licensee sells non‐alcoholic drinks to someone < 21 years, it is an administrative violation, which may result in a fine of up to $1500 for both the licensee and employee, and possible suspension or revocation of the licence (28‐A ME Rev. Stat §2074‐A and §2081 (2023)).

In Maine, advertising restrictions relating to promotion in retail settings apply to drinks ≥ 0.5% ABV, meaning non‐alcoholic drinks are not subject to these regulations. Outside of retail settings, advertising of standard alcoholic drinks may not target minors < 21 years, and outdoor advertising where children may be present, for example near a school, is prohibited [80, 81]. To our knowledge, there are no specific marketing restrictions for non‐alcoholic beverages.

### Minnesota

Minnesota has a mixed control and licence model (in which private sectors can distribute and sell alcohol) [80]. In Minnesota, alcoholic drinks are defined as any beverage containing > 0.5% ABV. Any product advertised as a non‐alcoholic drink is required to state that the product contains < 0.5% ABV on the label. If labelled correctly, the product would be regulated like a soft drink and therefore no licence is required to sell non‐alcoholic drinks. This means that non‐alcoholic drinks can be sold 24 h a day, 7 days a week. Minnesota does not have an age restriction, since non‐alcoholic drinks are regulated as if they were a soft drink.

In Minnesota, advertising using images of children in adverts of standard alcoholic drinks is prohibited [81]. The state also prohibits alcohol industry sponsorship of school events when the sponsorship involves sale or consumption of alcohol. Minnesota additionally prohibits the use of false, deceptive or misleading advertising [16]. To our knowledge, there are no specific marketing restrictions for non‐alcoholic beverages.

### New Jersey

New Jersey has a licence model [80]. In New Jersey, alcoholic drinks are defined as any fluid or solid capable of being converted into a fluid, suitable for human consumption, and having an alcohol content of > 0.5% ABV NJ Rev. Stat § 33:1–1 (2023). This means that non‐alcoholic drinks do not meet this definition and are therefore not regulated by the licensing legislation, and non‐alcoholic drinks can be sold without a licence, 24 h a day, 7 days a week. Non‐alcoholic drinks do not have age restrictions and can be sold to a person < 21 years. For a product to call itself alcohol‐free, it is required to have an alcohol content of 0% ABV.

In New Jersey, advertising of standard alcoholic drinks may not target minors and cannot use images that associate alcohol with athletic achievements [81]. New Jersey additionally prohibits use of false, deceptive or misleading advertising and advertising cannot be ‘lewd or obscene, or suggests that the use of any alcoholic beverage will result in health or athletic benefits’ [82]. Further, there cannot be improper use of religious symbols or reference to minors. To our knowledge, there are no specific marketing restrictions for non‐alcoholic beverages.

### Kentucky

Kentucky has a licence model [80]. In Kentucky, non‐alcoholic spirits, wine or beer are classified as alcoholic drinks (expert communication). As such, all rules and regulations concerning alcoholic drinks apply to their non‐alcoholic counterparts. Relatedly, it is an offence to sell non‐alcoholic beverages to minors < 21 years. The licensing legislation includes restrictions on the days and time alcohol can be sold, which includes non‐alcoholic drinks. This means that non‐alcoholic drinks, and their standard strength counterparts, can only be sold between 06.00 and 04.00 Monday to Saturday, and between 13.00 and 04.00 on Sunday (KY Rev. Stat § 113.40). In Kentucky, there are some dry counties where the sale of alcohol is prohibited, which would include the sale of non‐alcoholic drinks [83].

Regulation over in‐state electronic media is under the alcohol control agency's jurisdiction in Maine, which is considered a best practice (expert communication). Kentucky prohibits malt beverage advertising within 100 feet of schools and churches and false, deceptive, misleading, obscene or indecent advertising is also prohibited [84]. Based on the definition of alcohol, non‐alcoholic beverages would also seem to be subject to these regulations.
